# Living conditions, lifestyle habits and health among adults before and after the COVID-19 pandemic outbreak in Sweden - results from a cross-sectional population-based study

**DOI:** 10.1186/s12889-021-12315-1

**Published:** 2022-01-25

**Authors:** Anu Molarius, Carina Persson

**Affiliations:** 1 Centre for Clinical Research, Region Värmland, 651 85 Karlstad, Sweden; 2 Department of Public Health Sciences, Karlstad University, Karlstad, Sweden; 3 Department for Sustainable Development, Region Örebro County, Örebro, Sweden

**Keywords:** COVID-19, Health behaviour, Social factors, Health problems, Population studies

## Abstract

**Background:**

Studies on the public health consequences of COVID-19 pandemic showing data based on robust methods are scarce. The aim of this study was to investigate mental and physical health as well as living conditions and lifestyle habits in the general population before and after the COVID-19 outbreak in Sweden.

**Methods:**

The study is based on 2273 persons 16-84 years who responded to the national public health survey in February-May 2020 in Värmland county (overall response rate 45%). The differences between early respondents (before the outbreak, *n* = 1711) and late respondents (after the outbreak, *n* = 562) were studied using multivariate logistic regression, adjusting for background characteristics: age, gender, educational level, and country of birth. The same analyses were also completed in the corresponding survey carried out in February-June 2018.

**Results:**

Statistically significant differences between the groups were obtained for economic difficulties and worry about losing one’s job, which were more common among late respondents, and for sleeping difficulties, which were more common among early respondents after adjusting for background characteristics. There were no differences in other living conditions nor in lifestyle factors. Prevalence of good self-rated health, high blood pressure, aches in shoulders or neck, anxiety or worry and stress did not differ between the groups. In 2018, the only statistically significant difference between early and late respondents concerned economic difficulties.

**Conclusions:**

Very few differences in living conditions, lifestyle factors and health were observed in the study population before and after the COVID-19 outbreak. The results suggest that, in addition to a possible decrease in sleeping difficulties, the prevalence of being worried about losing one’s job increased among the employed after the outbreak.

**Supplementary Information:**

The online version contains supplementary material available at 10.1186/s12889-021-12315-1.

## Background

In March 2020, the worldwide pandemic of coronavirus causing COVID-19 reached Sweden. This led to restrictions in many sectors of society, an increased burden on health care, and an economic downturn with a sharp increase in layoffs and unemployment. The impact of the pandemic on the mental health of the population has not yet been investigated in depth in Sweden, but a few studies have been published. A longitudinal study on 1071 older adults, aged 65-71 years, found that mental well-being remained stable or was even higher in the early days of the pandemic compared to previous years [1]. A cross-sectional online survey of 1212 adult volunteers showed high levels of depression, anxiety, and insomnia but no comparison could be made with the situation before the COVD-19 outbreak [2]. An early study in the UK showed that the prevalence of depression and anxiety increased, especially among young adults, immediately after the first lockdown [3]. In Sweden, it was expressed that, based on past experiences of economic crises, it is likely that the COVD-19 pandemic will lead to a rise in mortality in the future [4]. This is because mass unemployment leads to increased mortality in the population, including mortality from alcohol-related diseases, suicide, and cardiovascular diseases, especially among men with low socioeconomic status [5].

The negative effects of the pandemic on mental health may arise from social measures such as quarantine and lockdown, fear of COVID-19 disease and lifestyle changes [6, 7]. In addition, some specific population groups such as young people, the elderly and people with learning disabilities and mental disabilities may be more affected than others [7]. The WHO and the UN have also highlighted the impact of the pandemic on the mental health of the population and the need to invest in health promotion and prevention in addition to health interventions [8, 9].

Mental health is strongly linked to the individual’s living conditions and lifestyle habits [10, 11]. The recommendations to the public made by the authorities due to the COVID-19 pandemic called on people to reduce their social contacts and those over 70 years of age were recommended to refrain from seeing people outside their own family. However, social relations are in many ways important for mental health [12]. Social support is a protective factor that can act as a buffer in psychosocial crisis situations or pressures. Furthermore, involuntary loneliness has been shown to have a strong link to depression among the elderly [13]. In addition to social relations, economic factors have a major impact on mental health. Those with financial difficulties have more mental health problems and depression [14, 15]. Recipients of financial assistance and unemployed young adults have a higher incidence of mental health problems than others [16]. Other factors strongly linked to mental ill-health are physical inactivity, daily smoking, and obesity [17–19].

Mental health is also associated with physical health. This is especially true for musculoskeletal disorders that are often work-related and symptoms of overexertion. Psychological factors such as stress and anxiety are also assumed to be related to musculoskeletal pain. Anxiety, nervousness, and experiences of mental stress increase muscle tension which contributes to pain, especially in the neck and shoulders [20].

Population surveys are commonly used to measure living conditions, lifestyle habits and health in the general population. The answers from persons who respond early or late, that is before and after a first reminder to a survey questionnaire tend, however, to differ in several ways [21]. For example, non-native persons, those with only pre-secondary education and younger age groups are more often late respondents. Furthermore, younger people and people with low levels of education can more often be reached by telephone follow-ups of non-respondents [22, 23]. We observed in a large survey in Sweden, that it was somewhat more common with good health as well as being physically active and having trust in others among those who responded early compared to those who responded late [21]. However, anxiety and nervousness were somewhat more common among those with late responses. These differences persisted even when age was taken into account.

A panel of experts on mental health in the UK published a recommendation for mental health research in Lancet Psychiatry in the context of the COVID-19 pandemic [24] and called for high-quality data and integration of different perspectives. Another group also emphasized the importance of monitoring mental disorders as well as risk factors such as unemployment, economic difficulties, alcohol consumption, and lack of social support in the population [25]. Several studies on the adverse effects of the pandemic on mental health and risk factors among adults, for example in Sweden [2], Canada [26] and Australia [27] were published after the first wave of the pandemic. However, these were usually conducted only after the outbreak of the pandemic and based on questionnaires distributed via social media, leading to self-selection, and have therefore probably exaggerated the effects of the pandemic [28]. Since then, many more studies have been published, including a review on the mental health effects of the pandemic [29] and a rapid review on the cardiovascular risk factors [30]. Neither of these reviews included, however, studies from Sweden. In addition, as Freiberg et al. [30] indicated, there is a high number of epidemiological studies on the impact of COVID-19 lockdown measures on modifiable cardiovascular risk factors, but only a few have used probability sampling methods. Epidemiologically robust methods, such as population studies based on random population sampling and the use of exactly the same questions before and after the outbreak of the pandemic are therefore of great value.

The aim of this study was to highlight mental and physical health as well as living conditions and lifestyle habits in the adult population before and after the COVID-19 outbreak in one county in Sweden by comparing early and late respondents to the public health survey “Health on equal terms?” carried out in February-May 2020.

## Methods

The study is based on data from the population survey “Health on equal terms?” conducted in collaboration with the Public Health Agency of Sweden [31]. The national survey started in 2004 and has been carried out every two years since 2016 to monitor the health of the population in Sweden. The age group addressed is 16–84 years. The sample frame is the total population register at Statistics Sweden, the statistical administrative authority in Sweden, covering all inhabitants in the country. The national simple random sample in 2020 included 40,000 persons.

The present study is based on data from one county (Värmland) where an extended simple random sample was drawn. In total, the questionnaire was sent to 5091 persons in the county and 2273 individuals answered the questionnaire giving an overall response rate of 45%. The questionnaire was postal but could also be answered online. Data collection was discontinued after two postal reminders. In Värmland county, the first COVID-19 cases were reported on 6th March 2020 [31]. To define those who replied before and after the COVID-19 outbreak in Sweden the respondents were divided into early (*n* = 1711) and late (*n* = 562) respondents, i.e. those responding between 3th February and 11th March 2020, and those responding between 12th March and 5th May, respectively. The date 11th March coincided with posting the first reminder of the survey.

Värmland county is situated in the west of Mid-Sweden, bordering to Norway, and comprises about 282,000 inhabitants. It includes one bigger city with over 90,000 inhabitants and 15 smaller municipalities. The incidence of COVID-19 was lower in Värmland than in Sweden in general during March-May 2020 and by the last week in May 533 persons had been diagnosed with COVID-19 in Värmland [31].

The measures taken to combat the COVID-19 pandemic in Sweden included e.g. recommendations to keep distance to other people, to wash hands often, to stay at home when having symptoms of flu, to avoid travelling abroad and unnecessary travelling in Sweden, to avoid public places with crowds, and to work from home when possible. Those over 70 years of age were recommended to refrain from seeing people outside their own family. In the end of March, public gatherings of more than 50 persons were forbidden. No total lockdown was, however, instituted in Sweden.

To explore whether the results observed in 2020 are due to the COVID-19 pandemic, the same analyses were run in the corresponding “Health on equal terms?” survey which was carried out between 28th February and 18th June 2018. In total, 2142 persons aged 16-84 years responded to the survey in Värmland county with an overall response rate 42%. Out of these, 1660 individuals responded before (early respondents) and 482 after (late respondents) the first reminder sent on 10th April 2018.

### Confounding variables

Information on gender, age, level of education and country of birth are based on register data from Statistics Sweden. Educational level was categorised into three levels: compulsory education, secondary education, and postsecondary education. Country of birth was dichotomized into those born in Sweden and those born outside Sweden.

### Outcome variables

#### Living conditions

Social support was measured with the question “Do you have anyone you can share your innermost feelings with and confide in?” (yes/no).

Economic difficulties were estimated with the question “During the last 12 months, have you ever had difficulty in managing the regular expenses for food, rent, bills etc.?”. The response options were “no”, “yes, once”, “yes, more than once” where the last two categories were combined to yes.

Trust in other people was measured with the question “Do you think that, in general, people can be trusted?” (yes/no). Employed people were defined as being worried about losing their job if they answered “yes” to the question “Are you worried about losing your job in the coming year?”

#### Lifestyle factors

Two questions for measuring physical activity were used. The first question was: How much time do you spend in a normal week on physical training that leaves you out of breath – for example running, fitness training, or ball sports? The response options were: 0 min/no time; less than 30 min; 30–59 min (0.5–1 h); 60–89 min (1–1.5 h); 90–119 min (1.5–2 h); 2 h or more. The second question was: How much time do you spend in a normal week on daily activities – for example walking, cycling, or gardening? Count all time together (at least 10 min at a time). The response options were: 0 min; less than 30 min; 30–59 min (0.5–1 h); 60–89 min (1–1.5 h); 90–149 min (1.5–2.5 h); 150–299 min (2.5–5 h); 5 h or more. These questions are used to measure whether the respondent reaches 150 activity minutes per week as recommended by the WHO. The number of minutes from the physical training and daily activities were summed together, with the number from the first variable counting double [32].

Sitting duration was asked with the question “How much do you sit during a normal day, not counting sleep?” The answer categories were dichotomised into those who sit less than 10 h and those who sit at least 10 h a day.

Smoking was measured using the question “Do you smoke” (“no”, “yes, sometimes”, “yes, daily”).

Alcohol consumption was measured using Alcohol Use Disorders Identification Test-C (AUDIT-C). AUDIT-C is a widely used and validated screening instrument of alcohol use. It comprises three questions on the frequency and quantity of alcohol consumption. We used the following cut-offs for risk-drinker: 6 or more points in men and 5 or more points in women [32].

#### Health

The following variables were used to measure the respondents’ health [32]. Self-rated health (SRH) was measured with the question “How would you describe your health in general?”. Response options were very good, good, fair, poor and very poor. In the statistical analysis the options were dichotomised into good (very good or good) and poorer than good (fair, poor or very poor) SRH.

Illnesses were measured with the following question: Do you have any of the following illnesses (with answer options No; Yes, but no discomfort; Yes, minor discomfort; Yes, severe discomfort)? Illnesses included high blood pressure, and the last three categories were combined to Yes.

Symptoms were derived from the question: Do you have any of the following discomforts or symptoms? These included “aches in the shoulders or neck”, “sleeping difficulties” and “anxiety or worry”. The answer categories were No; Yes, minor discomfort and Yes, severe discomfort, where the two latter categories were combined to Yes.

Stress was measured with the question “Do you feel stressed at present? By stressed, we mean a condition where you feel tense, restless, nervous, uneasy or unable to concentrate.” The answer options were Not at all; To some extent; Quite a lot and Very much, where the last three options were defined as having stress.

### Ethical considerations

The study followed the Swedish guidelines for studies in social sciences and humanities, in accord with the Declaration of Helsinki and the data are protected by the law of official statistics. The participants were informed that completed questionnaires would be linked to the Swedish official registries through personal identification numbers, to access registry information on gender, age, country of birth and educational level. The respondents thus gave their informed consent to the linking of registry data. The personal identification numbers were deleted before the data was delivered to Region Värmland. Statistics Sweden carried out the sampling, data collection and linkage with registry data and delivered the de-identified data. The study was approved by the Swedish Ethical Review Authority (Dnr 2020–04202).

### Statistical analysis

Differences in the distribution of background characteristics and SRH between early and late respondents were tested using chi-square statistics. Difference in mean age was tested using independent samples t-test. *P*-values < 0.05 were considered as statistically significant. The differences in living conditions, lifestyle habits, and health between early and late respondents were studied using multivariate binary logistic regression, with early/late response as the independent variable (reference category = early response), adjusting for background characteristics gender, age group, educational level, and country of birth. The results are reported as odds ratios (OR) and 95% confidence intervals (95% CI) for each living condition, lifestyle habit and health condition as outcome at a time. All analyses were conducted in IBM SPSS Statistics, version 26.

## Results

Table 1 shows the background characteristics of the study population among early and late respondents. Late respondents were younger than early respondents (mean age 53.5 and 58.2 years, respectively, *p* < .05) and they had a larger proportion of persons born outside Sweden than early respondents. There were no statistically significant differences in gender or level of education between the respondent groups.

**Table 1:** Background characteristics among early and late respondents in 2020, 16-84 years

	Total	Early respondent	Late respondent	*p*-value for difference between groups
N	2273	1711	562	
*Gender (%)*				.115
Women	53.2	54.2	50.4	
Men	46.8	45.8	49.6	
*Age group (%)*				<.001
16-29 years	10.9	9.7	14.6	
30-44 years	14.2	12.9	18.0	
40-64 years	32.1	31.6	33.6	
65-84 years	42.9	45.8	33.8	
*Educational level (%)*				.164
Low	42.7	42.9	42.2	
Medium	32.9	31.9	35.7	
High	24.4	25.2	22.0	
*Country of birth (%)*				.018
Sweden	89.2	90.1	86.5	
Other	10.8	9.9	13.5	

Some differences in living conditions, lifestyle factors and health between the two groups were observed (Table 2). Late respondents had more economic difficulties, had lower trust in other people, and were more often worried about losing their job than early respondents. There were no statistically significant differences between the groups regarding lifestyle factors. Sleeping difficulties were more common among early respondents whereas stress was more common among late respondents. Otherwise no statistically significant differences in health problems were found.

**Table 2:** Living conditions, lifestyle factors and health among early and late respondents 16-84 years in 2020 and adjusted odds ratios (with 95% confidence intervals in parenthesis) for living conditions, lifestyle factors and health among late respondents compared to early respondents

	Total	Early respondent	Late respondent	p-value for difference between respondent groups^a^	Adjusted OR^b^ (95% CI)
*Living conditions (%)*					
Economic difficulties	10.2	9.0	13.9	.001	**1.37 (1.02-1.86)**
Social support	90.3	90.4	90.0	.790	1.07 (0.77-1.49)
Trust in other people	79.3	80.5	75.8	.019	0.87 (0.69-1.11)
Worried about losing one’s job (employed)	10.6	8.6	16.0	<.001	**1.77 (1.19-2.64)**
*Lifestyle factors (%)*					
Physically active	64.6	65.6	61.6	.089	0.83 (0.67-1.02)
Sits at least 10 h/day	17.9	17.7	18.7	.576	0.97 (0.75-1.25)
Daily smoker	7.1	6.9	7.9	.424	1.14 (0.79-1.65)
Risk drinker	12.4	12.1	13.5	.373	1.04 (0.77-1.39)
*Health (%)*					
Good self-rated health	68.3	67.3	71.6	.057	1.18 (0.95-1.47)
Pain in shoulders or neck	52.2	52.0	52.7	.787	1.11 (0.91-1.35)
High blood pressure	33.3	34.4	30.1	.058	1.03 (0.81-1.30)
Sleeping difficulties	43.6	45.5	37.7	.001	**0.75 (0.61-0.92)**
Anxiety or worry	36.0	35.9	36.3	.870	0.95 (0.77-1.17)
Stress	51.1	49.4	56.4	.004	1.16 (0.94-1.42)

^a^ p-values are from chi-squared tests

^b^ Odds ratios adjusted for gender, age group, educational level, and country of birth. Outcome measures are living conditions, lifestyle factors, and health. Early/late response is the independent variable (reference category = early response). Statistically significant odds ratios marked with bold

Due to the differences in age and country of birth between the groups, multivariate logistic regression analyses were carried out adjusting for gender, age group, educational level, and country of birth (last column in Table 2). When adjusting for these background characteristics, statistically significant differences between early and late respondents remained for economic difficulties and worry about losing one’s job, which were more common among late respondents, and for sleeping difficulties, which were more common among early respondents. There were no differences in other living conditions nor in lifestyle factors. Self-rated health, high blood pressure, aches in shoulders or neck, anxiety or worry and stress did not differ between the groups when adjusting for background characteristics.

When the same multivariate logistic regression analyses were carried out in the corresponding survey in 2018, no statistically significant differences were observed between early and late respondents for lifestyle factors or health variables (see Supplementary Table S1, Additional file 1). For living conditions, the only statistically significant association was found for economic difficulties (OR: 1.64; 95% CI: 1.20-2.24). The prevalence of being worried about losing one’s job did not differ between early and late respondents (OR: 1.03; 95% CI: 0.64-1.65). The same applies to sleeping difficulties (OR: 0.84; 95% CI: 0.67-1.05).

## Discussion

In this study, very few differences were observed between early and late respondents in 2020 regarding living conditions, lifestyle factors and health. However, in the 2020-survey, it was more common among the late respondents to be worried about losing their job, and more common among the early respondents to report sleeping difficulties. These differences could not be seen in the corresponding survey in 2018.

The COVID-19 pandemic hit hard in Sweden in spring 2020 and until the last week in May 4499 people, predominantly persons over 70 years, had lost their lives and 2088 persons had been or were being treated in intensive care [31]. The short-term public health consequences of the COVID-19 pandemic and the restrictions related to it were however rather small at the population level in the present study. This is in line with the findings of the review of Prati et al. [29] who showed that the psychological impact of COVID-19 lockdowns was small in magnitude and highly heterogeneous, and no change for instance in social support was observed. Nevertheless, there can be subgroups where the impact has been detrimental. For example, Pierce et al. [33] found that even though the mental health of most UK adults remained resilient or returned to pre-pandemic levels between April and October 2020, about one in nine had a deteriorating or consistently poor mental health. Those in the deteriorating mental health group were more likely to be women, younger, without a partner, and have a previous mental illness [33]. The review of Freiberg et al. [30] reported, in turn, that physical activity decreased whereas sedentary behavior and alcohol consumption increased during the COVD-19 lockdown. But even though only studies using probability sampling were included in the review there were methodological shortcomings in many of these studies [30]. The only direct consequence of the pandemic in the present study seems to have been a rise in the proportion who worry about losing their job. Since worrying about losing one’s job is associated with mental health problems [9], it can be assumed that if this increase persists or continues, it will have a detrimental effect for the future mental health of the population. The proportion of persons having economic difficulties was also higher among the late respondents, but since similar findings were observed in the 2018 study, it is improbable that the difference in 2020 was due to the COVID-19 pandemic. Furthermore, this finding underlines the point that researchers should be observant and not to draw hasty conclusions that study results are due to the pandemic just because they occur during the same time period. Moreover, since early and late respondents differ from each other in many health-related aspects, it is important to adjust for known background characteristics and take the period of time into account.

The result that sleeping difficulties were more common among early than late respondents is somewhat puzzling. Similar findings were not observed in the 2018 study. An increase in the proportion who worry about losing their job suggests that an increase in sleeping difficulties may have been more likely. A cross-sectional study in Reggio Emilia in Italy, based on 1826 individuals, found that the first lockdown may have worsened the quality of sleep [34]. On the other hand, a study based on smartphone data in the US and 16 European countries found that the subjects increased their sleep duration but delayed their sleep onset during the COVID-pandemic in comparison to before the pandemic [35]. In Sweden, there was no total lockdown and the result that the mental well-being remained stable or was even higher after the outbreak of the pandemic among Swedish elderly [1] suggests that the changes in health are not necessarily always as expected. In addition, we did not find any increase in anxiety or worry or in stress. One, although somewhat improbable, explanation for the fact that late respondents reported less sleeping difficulties could be that an increasing number of employees were able to better control their working hours since they were working from home. This could perhaps contribute to better sleep. The decrease in sleeping difficulties may, of course, also be a spurious finding.

The response rate in our study was somewhat higher in 2020 (45%) than in 2018 (42%). This may have been due to the pandemic, increased interest in health issues, in recognizing the sender, the National Public Health Agency of Sweden, which had a press conference nearly every day at the beginning of the pandemic, or just because of chance.

Our sample was rather small, and we could not differentiate any groups within the two categories. It is possible that some groups have been affected by the COVID-19 pandemic more than others, for example those who have lost their jobs or persons over 70 years who were recommended to social isolation. It has been suggested that the pandemic will lead to increased inequalities in health [36]. In this study we were not able to assess health inequalities and changes in them. Also, the time frame was short and the effect of the second wave during the late autumn 2020 and other long-term consequences are not yet known. The survey was also limited to one county, where the number of cases treated in hospital care in relation to population was rather limited compared to for example the county of Stockholm [31]. The restrictions due to the pandemic were, however, similar.

The strength of our study is that it is based on a random population sample and the same questionnaire was used both by the early and late respondents. Another advantage is that we could compare the results directly with the survey carried out in 2018 so that false conclusions could be avoided. Several studies on the adverse effects of the pandemic on mental health and risk factors among adults have already been published [2, 26, 27]. However, it has been noted that these have usually been conducted only after the outbreak of the COVID-19 pandemic and been based on questionnaires distributed via social media, leading to self-selection and probable exaggerated effects of the pandemic [28–30]. Many papers on the pandemic have been published in public health journals, and even though more studies using robust methodology have been published since the early days of the pandemic, papers showing data based on robust methods have been scarce [29, 30, 37]. In addition, the results can vary between countries and the time of the study. For example, a study from Austria showed that COVID-19 restriction measures resulted in increased, but only on short-term, levels of loneliness among older adults during the lockdown [38].

## Conclusions

In conclusion, there was a statistically significant difference between early and late respondents in the study population in 2020 for worry about losing one’s job that could not be observed in the 2018-material. It is probable that this is attributable to the outbreak of the COVID-19 pandemic. The observed decrease in sleeping difficulties remains more puzzling. More research on the short- and long-term public health consequences of the pandemic in the general population and in different subgroups as well differences between populations, using robust data and methods, is thus needed.

## Supplementary Information

**Additional file 1: Table S1**. Living conditions, lifestyle factors and health among early and late respondents 16-84 years in 2018 and adjusted odds ratios (with 95% confidence intervals in parenthesis) for living conditions, lifestyle factors and health among late respondents compared to early respondents.

## Data Availability

The datasets generated and/or analysed during the current study are not publicly available due to confidentiality and regulations under the Swedish law (the Public and Privacy Act 2009: 400, Chapter 24, Section 8), but descriptive data in table form are available from the corresponding author on reasonable request.

## Abbreviations

OR: Odds ratio
CI: Confidence interval
AUDIT-C: Alcohol Use Disorders Identification Test-C
SRH: Self-rated health
WHO: World Health Organization
