# Supplementary material for: Living conditions, lifestyle habits and health among adults before and after the COVID-19 pandemic outbreak in Sweden - results from a cross-sectional population-based study
Source: BMC Public Health. 2022 Jan 25;22:171. doi: 10.1186/s12889-021-12315-1 (PMC8787439; doi:10.1186/s12889-021-12315-1)
Supplement: Supplementary file 1 — Additional file 1: Table S1. Living conditions, lifestyle factors and health among early and late respondents 16-84 years in 2018 and adjusted odds ratios (with 95% confidence intervals in parenthesis) for living conditions, lifestyle factors and health among late respondents compared to early respondents. [file 12889_2021_12315_MOESM1_ESM.docx]

Table S1. Living conditions, lifestyle factors and health among early and late respondents 16-84 years in 2018 and adjusted odds ratios (with 95% confidence intervals in parenthesis) for living conditions, lifestyle factors and health among late respondents compared to early respondents.

|  | Total | Early respondent | Late respondent | p-value for difference between respondent groups | Adjusted OR^1^ (95% CI) |
| --- | --- | --- | --- | --- | --- |
| *N* | 2,142 | 1,660 | 484 |  |  |
| *Living conditions (%)* |  |  |  |  |  |
| Economic difficulties | 10.4 | 8.8 | 16.0 | <.001 | **1.64 (1.20-2.24)** |
| Social support | 87.9 | 88.0 | 87.5 | .755 | 1.01 (0.73-1.39) |
| Trust in other people | 78.4 | 79.9 | 73.2 | .002 | 1.23 (0.96-1.58) |
| Worried about losing one’s job (employed) | 9.4 | 9.3 | 9.5 | .924 | 1.03 (0.64-1.65) |
|  |  |  |  |  |  |
| *Lifestyle factors (%)* |  |  |  |  |  |
| Physically active | 63.8 | 64.6 | 60.7 | .114 | 0.85 (0.68-1.06) |
| Sits at least 10 h/day | 15.5 | 15.5 | 15.6 | .960 | 0.87 (0.65-1.18) |
| Daily smoker | 6.6 | 6.1 | 8.4 | .086 | 1.37 (0.92-2.04) |
| Risk drinker | 14.9 | 14.6 | 15.7 | .557 | 0.96 (0.71-1.30) |
|  |  |  |  |  |  |
| *Health (%)* |  |  |  |  |  |
| Good self-rated health | 69.5 | 70.1 | 67.5 | .294 | 0.82 (0.65-1.04) |
| Pain in shoulders or neck | 52.6 | 53.4 | 49.9 | .180 | 0.87 (0.70-1.08) |
| High blood pressure | 28.9 | 29.6 | 26.7 | .220 | 1.17 (0.90-1.51) |
| Sleeping difficulties | 38.8 | 39.8 | 35.4 | .085 | 0.84 (0.67-1.05) |
| Anxiety or worry | 36.4 | 35.8 | 38.6 | .262 | 1.04 (0.83-1.30) |
| Stress | 51.4 | 49.9 | 56.5 | .011 | 1.12 (0.90-1.39) |
|  |  |  |  |  |  |

^1^ Odds ratios adjusted for gender, age group, educational level, and country of birth. Outcome measures are living conditions, lifestyle factors, and health. Early/late response is the independent variable (reference category=early response). Statistically significant odds ratios marked with bold.
